# Early COVID‐19 testing is critical to end the pandemic

**DOI:** 10.1002/jgf2.420

**Published:** 2021-01-28

**Authors:** Kazuki Shimizu, Keita Kondo, Yasuhiro Osugi, Masashi Negita, Hiromi Mase, Taro Kondo, Makoto Aoki, Kiyosu Taniguchi, Kenji Shibuya, Yasuharu Tokuda

**Affiliations:** ^1^ Department of Health Policy London School of Economics and Political Science London UK; ^2^ Faculty of Public Health and Policy London School of Hygiene and Tropical Medicine London UK; ^3^ Department of Community Based Medicine Fujita Health University Toyoake Japan; ^4^ Department of General Internal Medicine Toyota Regional Medical Center Toyota Japan; ^5^ Department of Surgery Tosei General Hospital Seto Japan; ^6^ Department of Epidemiology and Public Health Institute of Epidemiology and Health Care University College London London UK; ^7^ Kondo Clinic Tokyo Japan; ^8^ Sakura Seiki, Co. Tokyo Japan; ^9^ National Hospital Organization Mie National Hospital Mie Japan; ^10^ Institute for Population Health King's College London London UK; ^11^ Muribushi Okinawa Center for Teaching Hospitals Okinawa Japan

## 1 THE ROLE OF TESTING IN MEDICINE AND PUBLIC HEALTH

Testing is critical to break transmission chains of severe acute respiratory syndrome coronavirus 2 (SARS‐CoV‐2) and end the coronavirus disease 2019 (COVID‐19) pandemic, but there are slight differences in its roles between medicine and public health. In medicine, protecting the health of the individual through prevention, diagnosis, and treatment is prioritized. Public health mainly focuses on improving population health and tries to promptly detect suspected outbreaks, prevent and control their spread, and enhance the surveillance system. The European Centre for Disease Prevention (ECDC) summarizes five objectives of testing: (a) to control transmission; (b) to monitor SARS‐CoV‐2 transmission rates and severity; (c) to mitigate the impact of COVID‐19 in healthcare and social care settings; (d) to detect clusters or outbreaks in specific settings; and (e) to maintain COVID‐19 elimination status once achieved.^1^

To meet the first objective, all individuals with suspected COVID‐19 symptoms must be promptly identified and tested after the onset of their symptoms. This is recommended by many public health agencies or other organizations at the global scale and practiced accordingly (Table 1). In medical perspectives, early detection of COVID‐19 can contribute to preventing sudden death as the pathogenesis of silent hypoxia has not been clarified. Considering an extended duration for virus shedding under specific circumstances, including immunocompromised patients, testing will also help recognize their viral load and clinical course. Moreover, diagnosing patients as COVID‐19 may eventually ensure that patients who suffer from long COVID can access to the social and medical support. Regarding advantages of testing in public health, detecting additional sporadic cases and clusters, in line with timely isolation, rigorous contact tracing, and quarantine, prevents further transmission. Besides, enhanced population‐based surveillance is vital to achieve the second objective and inform the public health decision. Healthcare workers (HCWs) in both primary healthcare and hospital settings are expected to be involved in this process.^1^

**TABLE 1:** RT‐PCR test indication among symptomatic patients with suspected COVID‐19 in selected organizations

Organization	Recommendation
Outside Japan	
World Health Organization	Anyone with symptoms should be tested, wherever possible.
Centers for Disease Control and Prevention, United States	People who have symptoms of COVID‐19.
European Centre for Disease Prevention and Control	Ideally, all people with COVID‐19 symptoms should be tested as soon as possible after symptom onset.
Ministry of Health, Ontario, Canada	Testing should be offered to, or arranged for, all patients with new or worsening symptoms that are compatible with COVID‐19 where possible and appropriate. Along with a different diagnosis (eg, bacterial infection), COVID‐19 testing should be considered in parallel if warranted.
British Columbia Centre for Disease Control, Canada	Recommend testing if any ONE of the following symptoms more predictive or strongly associated with COVID‐19 is reported: fever or chills; new onset or worsening of chronic cough; loss of sense of smell or taste; difficulty breathing.
Japan	
Japan Medical Association	COVID‐19 would be suspected after excluding infections by common respiratory pathogens such as bacteria, aspiration‐related organisms, *Mycoplasma*, *Chlamydophila*.
Japan Primary Care Association	Tests are considered for the following symptomatic patients: close contact history; high‐risk patients; moderate to severe symptoms; mild symptoms for at least 4 days.

Abbreviations: COVID‐19, coronavirus disease 2019; RT‐PCR, reverse transcription‐polymerase chain reaction.

The reference numbers of these organizations are shown in parentheses: World Health Organization.^8^ Centers for Disease Control and Prevention.^9^ European Centre for Disease Prevention and Control.^10^ Ministry of Health, Ontario, Canada.^11^ British Columbia Centre for Disease Control, British Columbia, Canada.^12^ Japan Medical Association.^13^ Japan Primary Care Association.^14^

## 2 A PUZZLING CONTROVERSY IN JAPAN: ACCESS TO TESTING AND PERFORMANCE OF RT‐PCR TEST

While many countries gravely expanded the testing capacity for diagnosing COVID‐19, citizens’ access to testing had not been adequately ensured in Japan.^2^, ^3^ Although the infectiousness among asymptomatic and presymptomatic individuals had been recognized in earlier phase, patients suffered from COVID‐19–related symptoms were generally instructed to stay home for 4 days in early 2020.^2^ Even after lifting the state of emergency in May, Japan made insufficient efforts to ramp up its testing capacity and logistics, and some professional societies still argue that self‐isolation at home must be prioritized compared with early testing (Table 1).

In addition, there has been a puzzling discussion regarding the performance of reverse transcription‐polymerase chain reaction (RT‐PCR) test. The nucleic acid amplification tests (NAATs), including RT‐PCR test, are the gold standard for diagnosis, and 100% specificity has been a scientific consensus;^4^ however, its specificity was perplexingly downgraded as low as 99% or 99.9% without presenting any solid evidence. False‐positive COVID‐19 results were criticized in a nonscientific manner,^3^ and scant discussion addressed the importance of external quality assessment schemes from the third party, which are necessary to prevent technical errors and reagent or laboratory contamination.^5^ Scientific evidence, and COVID‐19 data reported in Australia, New Zealand, China, and several other countries that achieved COVID‐19 elimination or containment could evidently reject this factitious argument.^3^, ^6^ Regarding sensitivity, there are several reasons to bring negative results among infected individuals, and RT‐PCR test has been clinically used to rule‐in COVID‐19 cases.^5^ Nevertheless, negative results were excessively conceptualized as whether investing in public health infrastructure and bolstering COVID‐19 tests will bring several disadvantages. Lack of knowledge and critical thinking among some HCWs, absence of their independent drive to learn from the multidisciplinary science, insufficiency of scientific journalism, and weakness in health communication^2^ perplexingly brought the distorted science to the public.

## 3 PHYSICIANS’ ACCOUNTABILITY DURING HEALTH EMERGENCY

To combat against the COVID‐19 pandemic, building a consolidated command and control system is vital. Presenting a clear grand strategy based on excellent science will help front‐line HCWs, including physicians, to correctly understand their roles in both medicine and public health, and successfully run find‐test‐trace‐isolate‐support systems.

First and foremost, physicians need to change their mindset and remove their biases. As the infectiousness of COVID‐19 starts before the onset of symptoms,^7^ all patients with suspected COVID‐19 symptoms, even milder cases, must be promptly tested. This will protect patients’ health and break transmission chains. RT‐PCR testing is the gold‐standard diagnostic test; therefore, it must be noted that joining the pseudoscience is completely contradictory to physicians’ professionalism. As every testing helps to end the pandemic, ensuring citizens’ access to testing is critical. Launching and managing test centers to the entire nation and strengthening logistics for postal testing service will ease citizens’ psychological barriers to testing and help detect more asymptomatic and presymptomatic COVID‐19 patients with transmissibility. These opportunities will accelerate COVID‐19 screening among essential workers who work in high‐risk environments. HCWs need to embrace people who have cooperated with COVID‐19 testing, rather than just ask citizens to comply with additional personal hygiene practices.

Second, HCWs must continuously learn from excellent science, regularly update their knowledge, critically appraise literature, review previous fallacies, and incorporate latest insights. The ongoing COVID‐19 pandemic explicitly suggested that policymakers and even some scientists sometimes ignored, cherry‐picked, and distorted science. Japan has been no exception.^2^ Without examining previous errors and getting appropriate feedback, the same mistakes will be repeated, thus magnifying patients’ risk.

The COVID‐19 pandemic in Japan elucidated that some physicians were not conscious of their roles for improving and promoting public health, as specified in Article 1 of the Medical Practitioners' Act. Physicians must note that solely appreciating medical perspectives and downgrading public health viewpoints are contradictory to their professionalism. As guaranteeing citizens’ access to testing and their social security during the isolation/quarantine period will drastically change the landscape, and early containment of COVID‐19 will contribute to protecting vulnerable populations and maintaining essential health services, physicians must be responsible for learning from previous errors, critically appraising the excellent science, and protecting patients from misinformation.

## CONFLICT OF INTEREST

We declare no competing interests.
